# Supplementary material for: Yeast cell fate control by temporal redundancy modulation of transcription factor paralogs
Source: Nat Commun. 2021 May 25;12:3145. doi: 10.1038/s41467-021-23425-0 (PMC8149833; doi:10.1038/s41467-021-23425-0)
Supplement: Supplementary file 1 — Supplementary Information [file 41467_2021_23425_MOESM1_ESM.pdf]

Supplementary Fig. 1

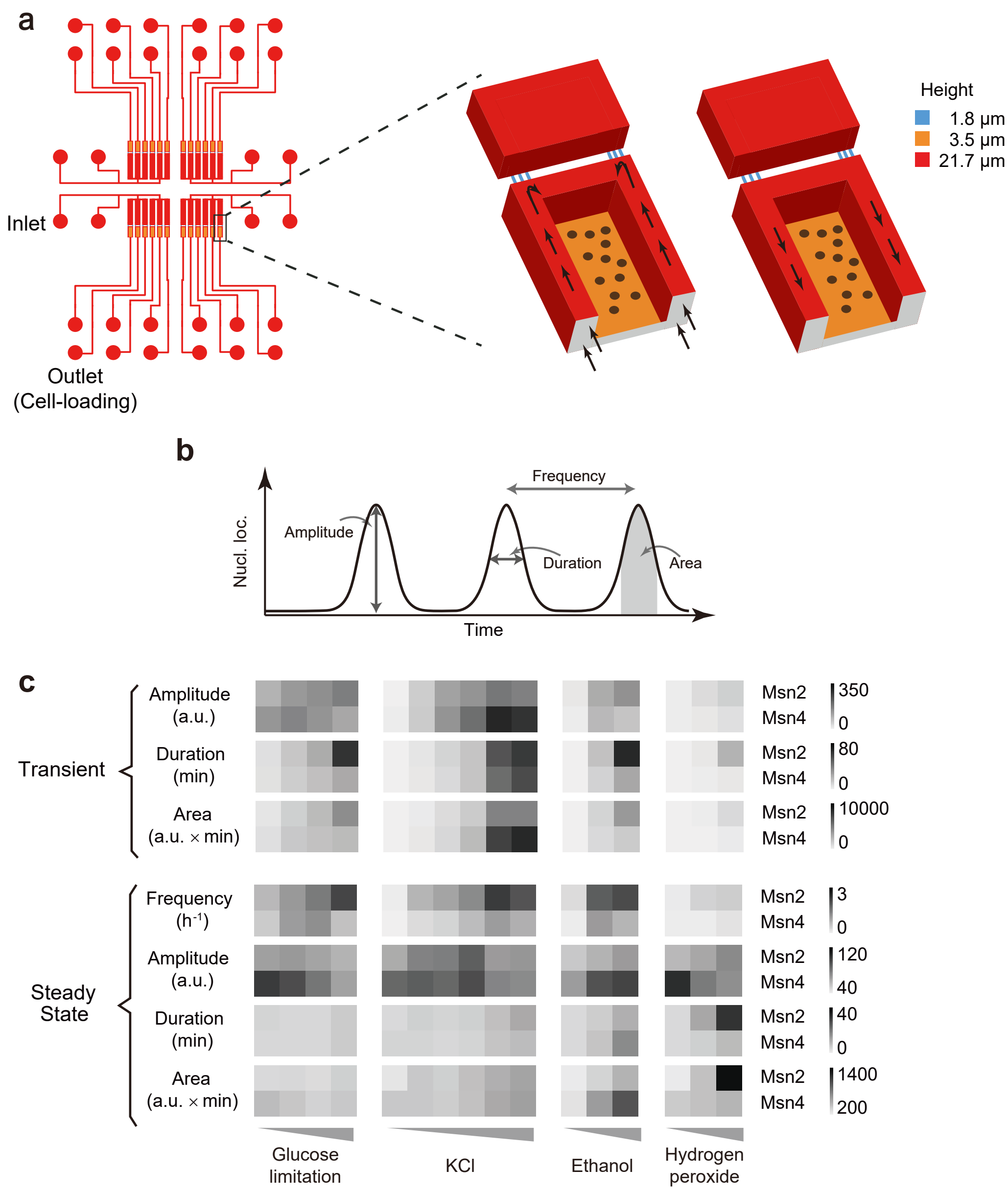

Supplementary Fig. 1: Systematic characterizations of Msn2 and Msn4 dynamics under various natural stresses in *S. cerevisiae* with microfluidics. (**a**) Design of the microfluidic chip[1]. A single chip can be simultaneously used for 4 separate stress conditions, each of which has 2 inlets for media switch and 6 outlets that are also used for cell loading. The right panels show a zoomed-in view of the imaging chamber. Cells were loaded from the outlets through the red channels and were pushed into the orange observation chamber due to the blockage of the blue fence for single-layer microscopy. Cells outside the observation chambers were later washed out by media from the inlets (right panel). (**b**) During stress responses, different characteristics of the pulsatile dynamics (e.g., amplitude, frequency, duration) could be modulated by the cell (schematic). (**c**) Modulation of different pulse characteristics for Msn2 and Msn4 during both transient and steady-state responses under various stresses, including glucose limitation stress (glucose concentration: 0.5%, 0.1%, 0.05%, and 0.01%, concentration for normal culture is 2%), osmotic stress (KCl concentration: 0.1 M, 0.25 M, 0.5 M, 0.8 M, 1 M, and 1.15 M), ethanol stress (ethanol concentration: 2%, 4%, and 6%), and oxidative stress (hydrogen peroxide concentration: 0.1 mM, 0.25 mM, and 0.5 mM).

Supplementary Fig. 2

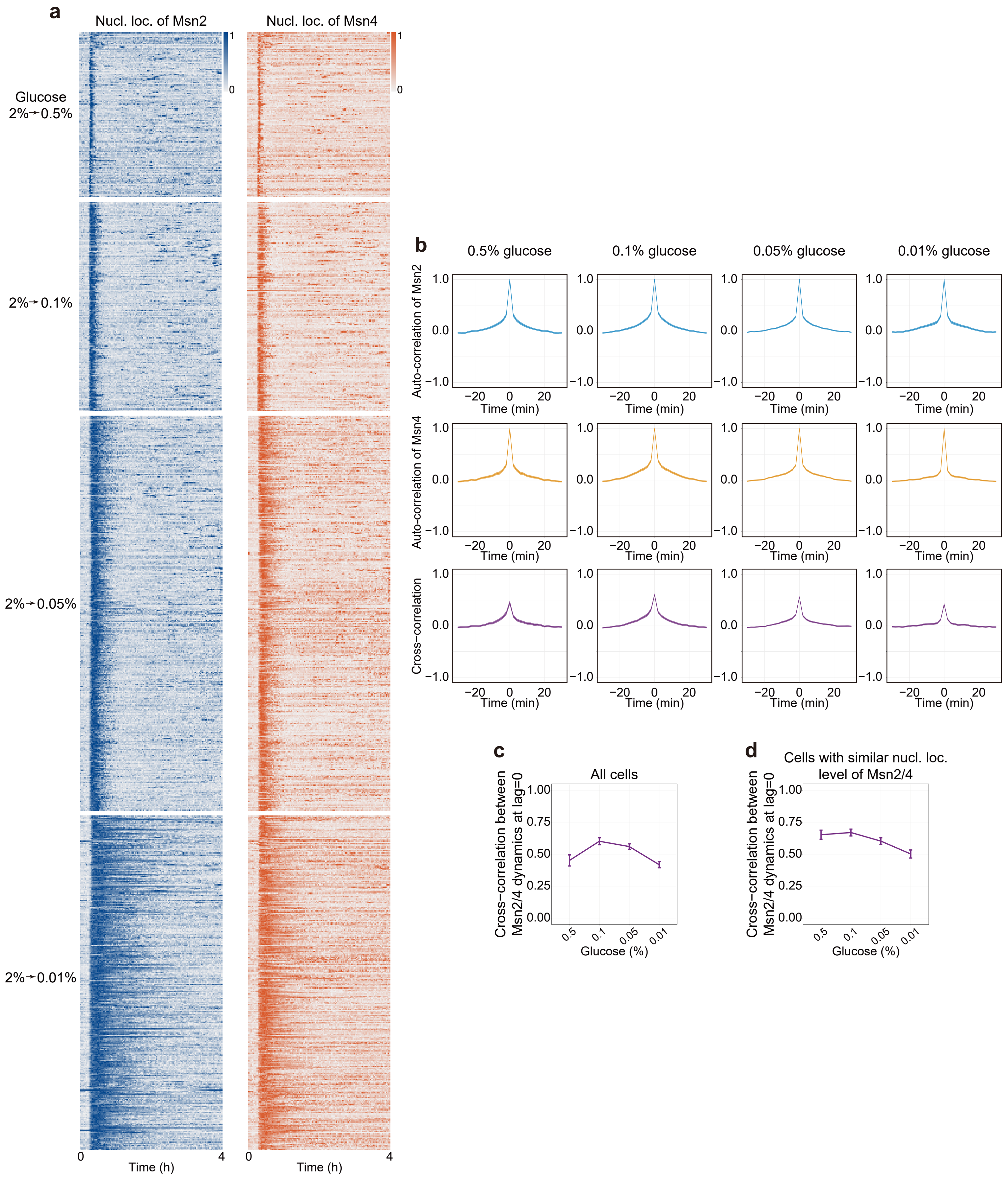

Supplementary Fig. 2: Pulsatile dynamics of Msn2 and Msn4 in *S. cerevisiae* are non-periodic and stochastic. (**a**) Single-cell trajectories of Msn2 and Msn4 nuclear localization under four glucose limitation conditions. Cells were subjected to a media switch from normal condition (2% glucose) to four levels of glucose limitation stress (0.5%, 0.1%, 0.05%, and 0.01% glucose), and the nuclear localizations of Msn2 and Msn4 were recorded and quantified. Each row represents one cell, and each trajectory of Msn2 (or Msn4) is normalized (min-max). (**b**) Auto-correlation analysis of Msn2/4 (blue/orange) dynamics and cross-correlation analysis between Msn2 and Msn4 (purple) dynamics from the same cells during steady-state responses. Shading indicates 95% CI and centers indicate means. (**c**) Cross-correlation in (**b**) at time lag zero. Error bars indicate 95% CIs and centers indicate means. (**d**) Cross-correlation at time lag zero for cells with comparable nuclear localization intensities across stress levels. To avoid the effect of pulse frequency on the calculation of cross-correlation between Msn2 and Msn4 dynamics, we sampled cells whose mean Msn2/4 nuclear localization intensities were between the first and the third quartile of all cells. Cross-correlation analysis as in (**c**) was performed for the chosen cells. Cell numbers are 39, 61, 91, and 69 for each stress level (from left to right). Error bars indicate 95% CIs and centers indicate means.

Supplementary Fig. 3

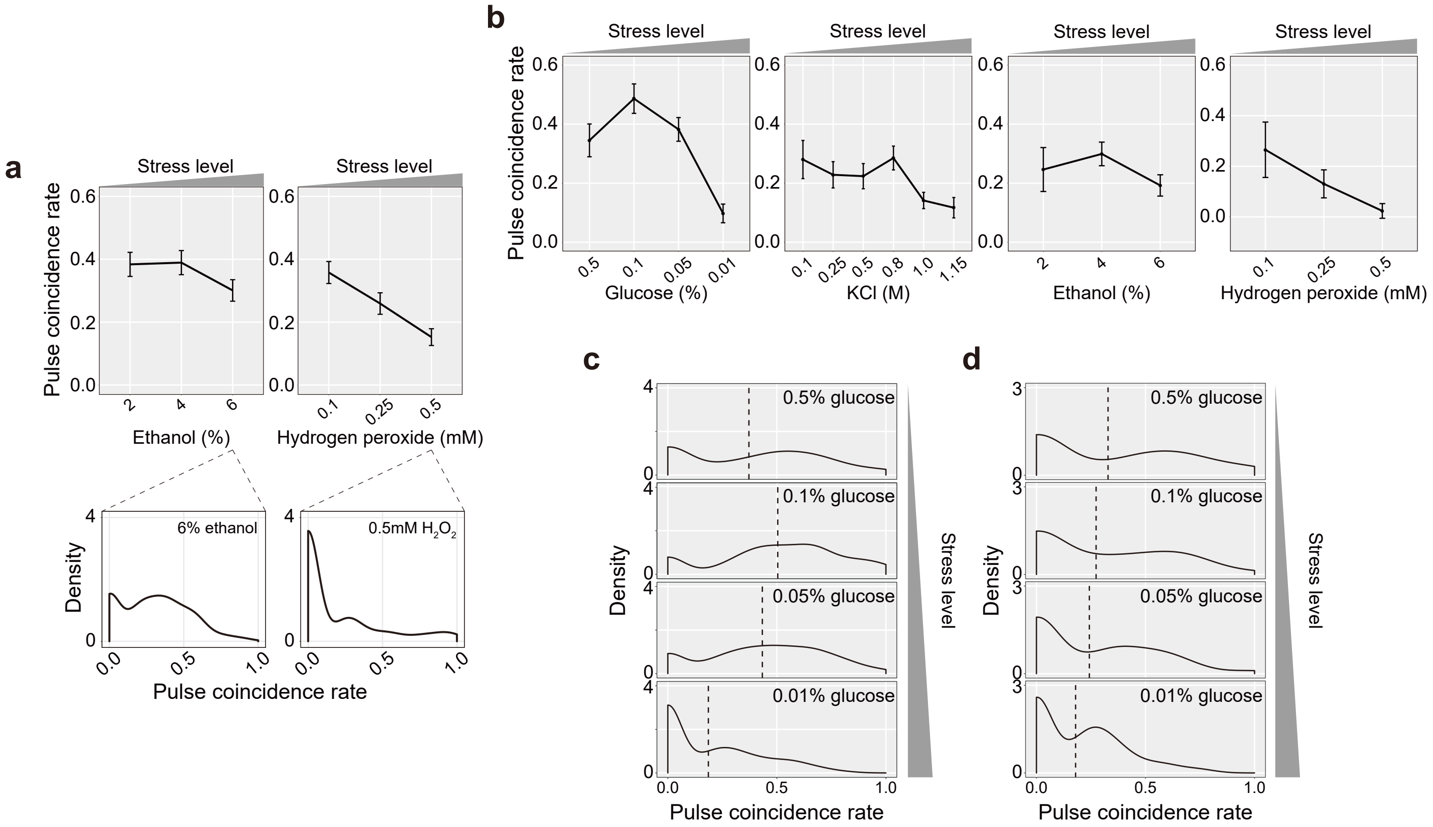

Supplementary Fig. 3: Characterizations of the modulation of temporal relationship between Msn2 and Msn4 under natural stresses in *S. cerevisiae*. (**a**) Pulse coincidence rate between Msn2 and Msn4 decreases as the level of ethanol or oxidative stress increases. Cell numbers are 54, 159, and 226 for ethanol stress conditions, and 67, 93, and 140 for oxidative stress conditions. Error bars indicate 95% CIs and centers indicate means. Pulse coincidence rate shows a bimodal distribution among single cells under the same stress condition (lower panels). (**b**) Pulse coincidence rate without gating pulse frequency (i.e., raw pulse coincidence rate) decreases as the stress level increases for all stress types tested. Cell numbers are 139, 176, 333, and 282 for glucose limitation stresses, 148, 182, 207, 181, 195, and 201 for osmotic stresses, 54, 159, and 226 for ethanol stresses, and 67, 93, and 140 for oxidative stresses. Error bars indicate 95% CIs and centers indicate means. (**c**) Distributions of single-cell pulse coincidence rate under different levels of glucose limitation stress. Dashed lines indicate means. (**d**) The bimodality in pulse coincidence rate distribution is not due to differences in pulse frequencies. To avoid the potential effects of pulse frequencies, we classified single cells as having high or low pulse coincidence rate and the same number of cells were then selected from both groups with the criteria that the relative differences in pulse frequencies are no more than 25%. Distributions of pulse coincidence rates for the two resulting groups of cells still display bimodality. Cell numbers are 60, 34, 80, and 90 (top to bottom).

Supplementary Fig. 4

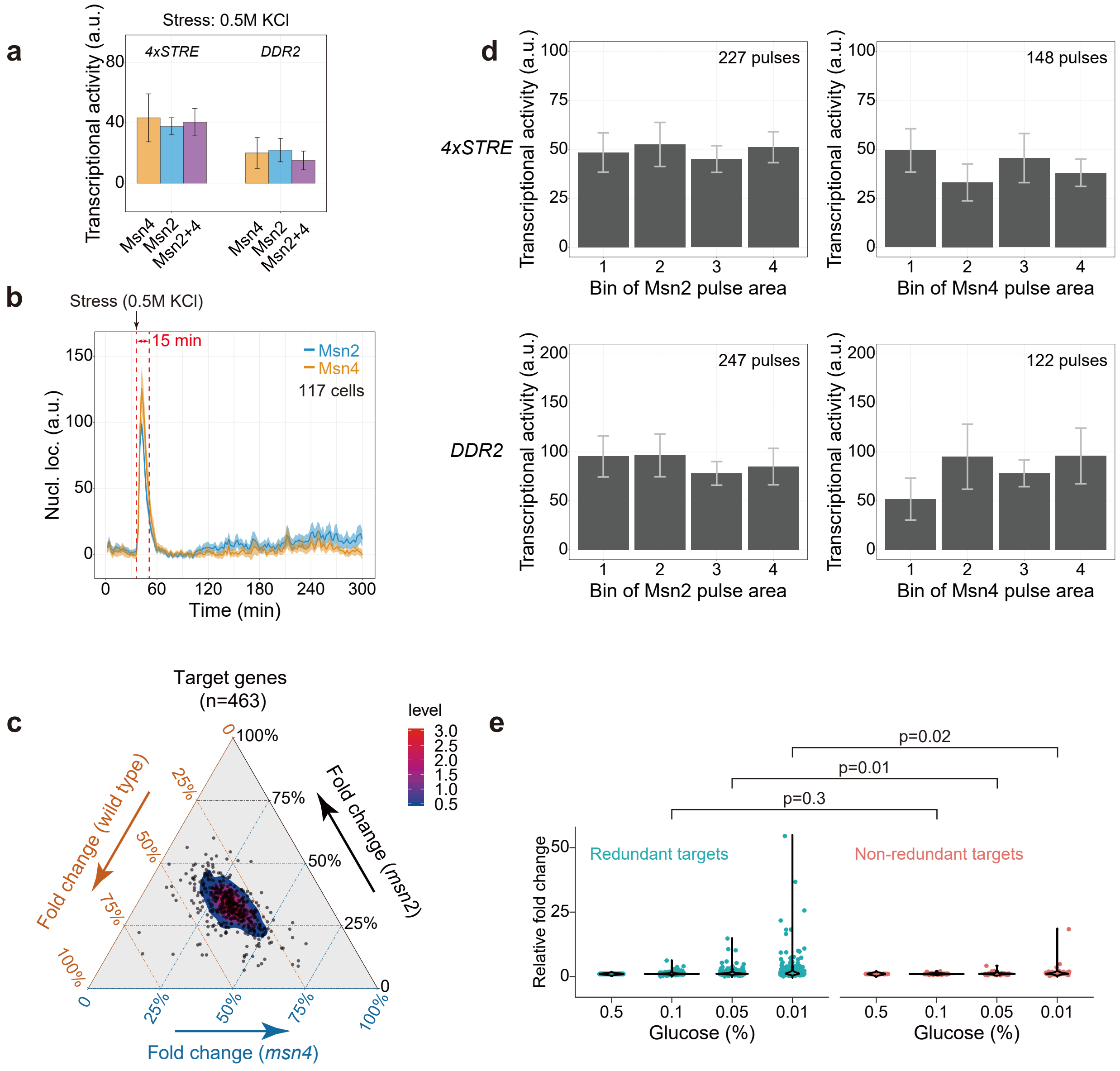

Supplementary Fig. 4: Characterizations of functional redundancy between individual Msn2 and Msn4 pulses. (**a**) Pulse-triggered averaging analysis of the transcriptional activities of PP7-based reporter genes (driven by either synthetic *4xSTRE* or natural *DDR2* promoter) in response to three types of TF dynamics under salt stress (0.5M KCl). Event numbers are 27, 289, 115, 25, 106, and 68 (from left to right respectively). Error bars indicate 95% CIs and centers indicate means. See also Supplementary Fig. 14b. (**b**) Average trajectories of Msn2 and Msn4 in wild type during response to sudden salt stress (0.5M KCl). Synchronized Msn2/Msn4 co-pulse across cell population was observed. Shading indicates 95% CI and centers indicate means. (**c**) Results from a biological replicate of Fig. 2c. (**d**) Transcriptional activities of PP7-based reporter genes for different doses of Msn2 or Msn4 pulses. Msn2-only and Msn4-only pulses in Fig. 2b were binned by pulse area (from small to large) and each bin has equal number of pulses. Transcriptional activities of PP7-based reporter genes (*4xSTRE* or *DDR2*) were calculated for each bin. Error bars indicate standard errors and centers indicate means. See also Supplementary Fig. 14c. (**e**) Results from a biological replicate of Fig. 3b. p values indicate one-sided K-S test.

Supplementary Fig. 5

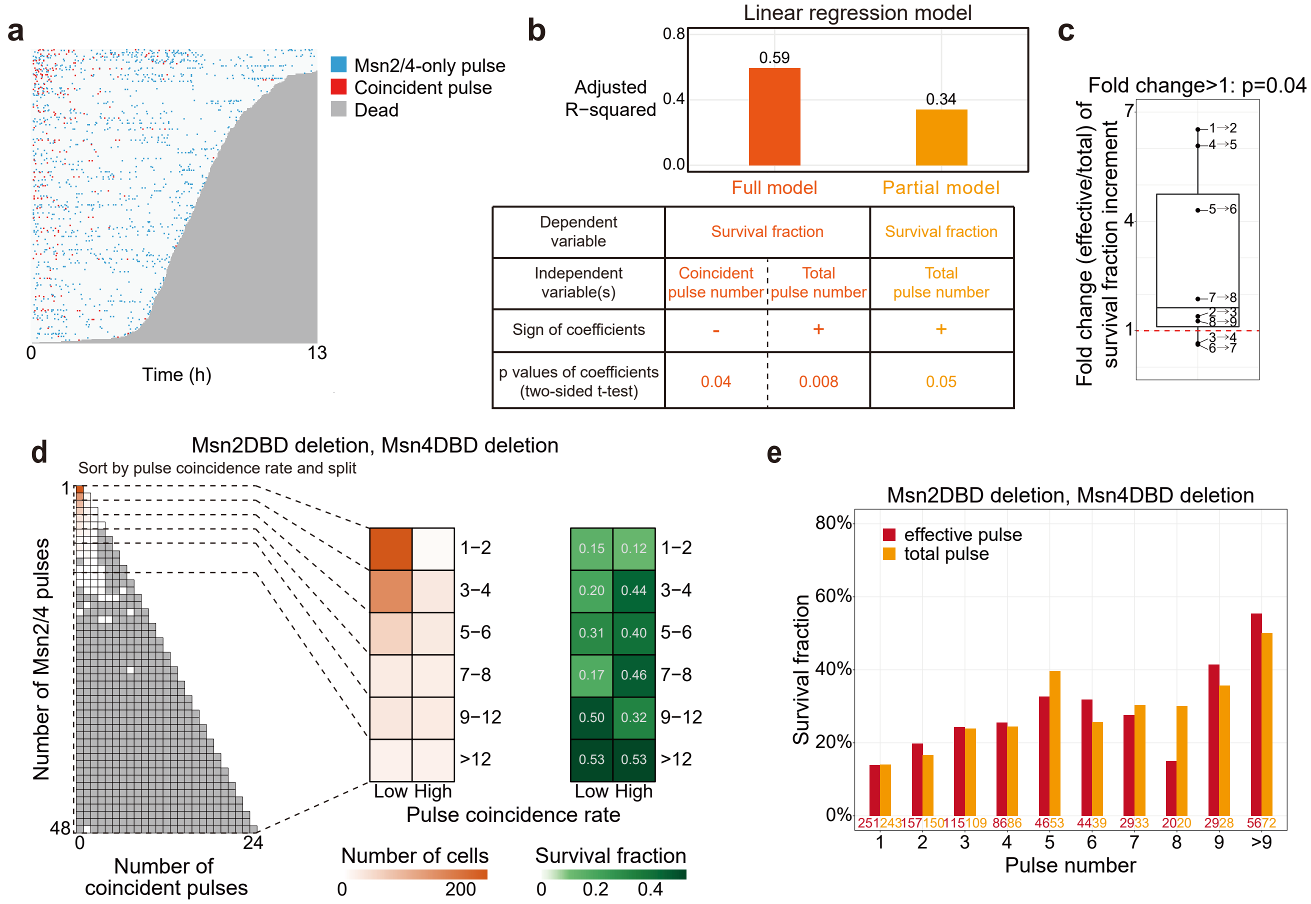

Dependent variable	Survival fraction		Survival fraction
Independent variable(s)	Coincident pulse number	Total pulse number	Total pulse number
Sign of coefficients	-	+	+
p values of coefficients (two-sided t-test)	0.04	0.008	0.05

Supplementary Fig. 5: Characterizations of the effect of temporal redundancy modulation on yeast stress survival. (**a**) Heat map of single-cell trajectories of 400 randomly sampled cells (out of 1676 cells) under glucose limitation stress (0.05% glucose). Cell death was identified by the DRAQ7 signal (Fig. 3c). Cells were vertically sorted by their time of death. Note that the symbols for pulses were slightly enlarged for clarity. (**b**) Statistical analysis showing that a linear regression model including the temporal relationship between Msn2 and Msn4 can better explain cell survival capacity with much higher adjusted R-squared (adjusted for the number of predictors in the models). Also, cell survival rate negatively correlates with the number of coincident pulses in the full model. Specifically, cells in Fig. 3d were binned by two dimensions (namely, total pulse number and coincident pulse number) and bins with greater than or equal to 20 cells were used for analysis. Dependent variable, independent variable(s), sign of coefficients and p values of coefficients (two-sided t-test) are shown in the table. (**c**) Adding an extra effective pulse yields a larger increase in cell survival capability compared to adding an extra total pulse in most scenarios. Fold changes of survival fraction increment between adding an extra effective pulse and adding an extra total pulse from each pulse number in Fig. 3e were calculated and shown as box plot. The box and horizontal line within the box represent interquartile range and median, the upper whisker extends from the box to the largest value no further than $1.5 \times$ IQR (where IQR is the inter-quartile range) and the lower whisker extends from the box to the smallest value at most $1.5 \times$ IQR. p value indicates one-sided Wilcoxon signed-rank test. (**d**) Transcriptional activities of Msn2 and Msn4 are necessary for the stress survival advantages for the cells with low temporal redundancy. We created a mutant strain where Msn2 and Msn4 do not contain their DNA binding domains (DBD) and performed similar experiments and analysis as in Fig. 3d. In this mutant strain, cells with low pulse coincidence rate do not have higher survival capacity as shown in Fig. 3d. Analysis similar to (**b**) on this dataset did not show a negative coefficient of coincident pulse number as in (**b**). (**e**) For cells with the same number of effective versus total Msn2/4 pulses in the mutant strain, the contrast in survival capability between the two groups as in Fig. 3e is abolished. Cell numbers are indicated below the bars.

Supplementary Fig. 6

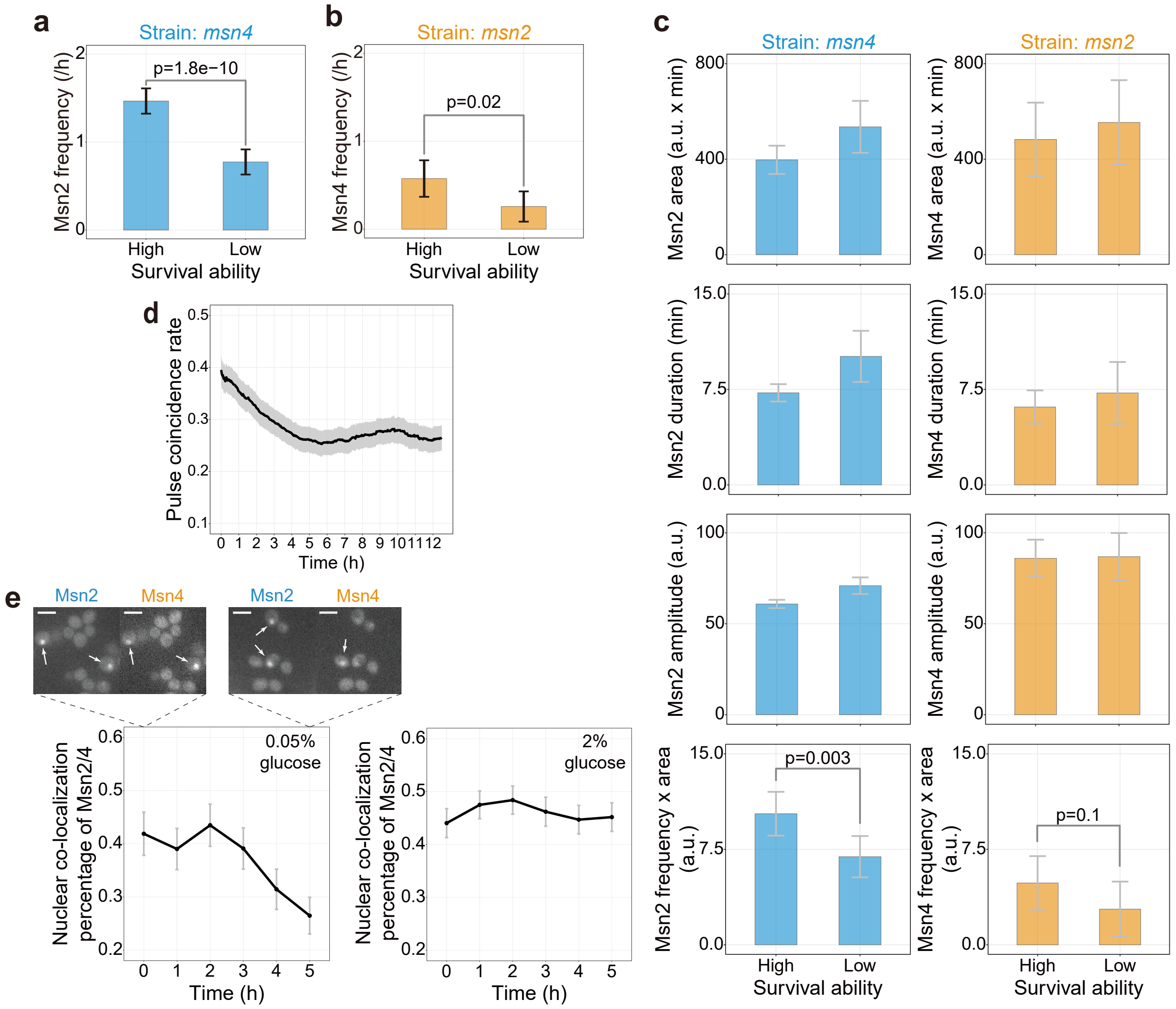

Supplementary Fig. 6: Additional experimental results supporting the effect of temporal redundancy modulation on yeast stress survival. a-b, Cells with higher Msn2 or Msn4 pulse frequency display higher stress survival ability. Similar assay as in Fig. 3d was performed for *msn4* (**a**) or *msn2* (**b**) strain under glucose limitation stress (0.05% glucose). The frequency of Msn2 in *msn4* (**a**) or Msn4 in *msn2* (**b**) strain during the first 8 hours of stress was calculated for each cell and cells were classified as having high or low survival ability depending on whether they survived additional 3 hours of stress. The average pulse frequency for each group was calculated and one-sided student's t-test was conducted. Error bars indicate 95% CIs. Cell numbers are 190 and 44 for (**a**) and (**b**), respectively. See also Supplementary Fig. 14d-e. (**c**) Msn2/4 pulse area/duration/amplitude are not positively correlated with survival ability, and increasing the nuclear accumulation (namely, pulse frequency times area) of Msn2 or Msn4 does not lead to enhanced cell survival as significantly as increasing pulse frequency alone. The average pulse area, duration, amplitude, and frequency times area for groups in (**a**) and (**b**) were calculated. Error bars indicate 95% CIs and p values indicate one-sided student's t-test. Cell numbers are 190 and 44 for *msn4* and *msn2*, respectively. See also Supplementary Fig. 14f. (**d**) Pulse coincidence rate between Msn2 and Msn4 along the time course under glucose limitation stress (0.05% glucose). For each time point, pulse coincidence rate between Msn2 and Msn4 before the time point was calculated for surviving cells based on the same method as in Fig. 1c. Cell number is 1672 at the start. Shading indicates 95% CI and centers indicate means. Note that such a change is likely due to the decrease in glucose concentration with time and the photo-toxicity. (**e**) Msn2/4 nuclear co-localization percentages over time under low glucose condition (0.05% glucose) or high glucose condition (2% glucose). Cells were cultured under either low or high glucose condition, and images were taken once per hour to quantify nuclear localizations of Msn2/4 as well as cell survival (based on DRAQ7). See Methods for details. Error bars indicate 95% CIs and centers indicate means. Cell numbers are 5263, 7611, 10271, 14602, 18975, and 21736 for 0.05% glucose, and 3191, 4506, 6661, 9178, 12330, and 18041 for 2% glucose. Example cropped snapshots are shown on the top and scale bars indicate 5 μm.

Supplementary Fig. 7

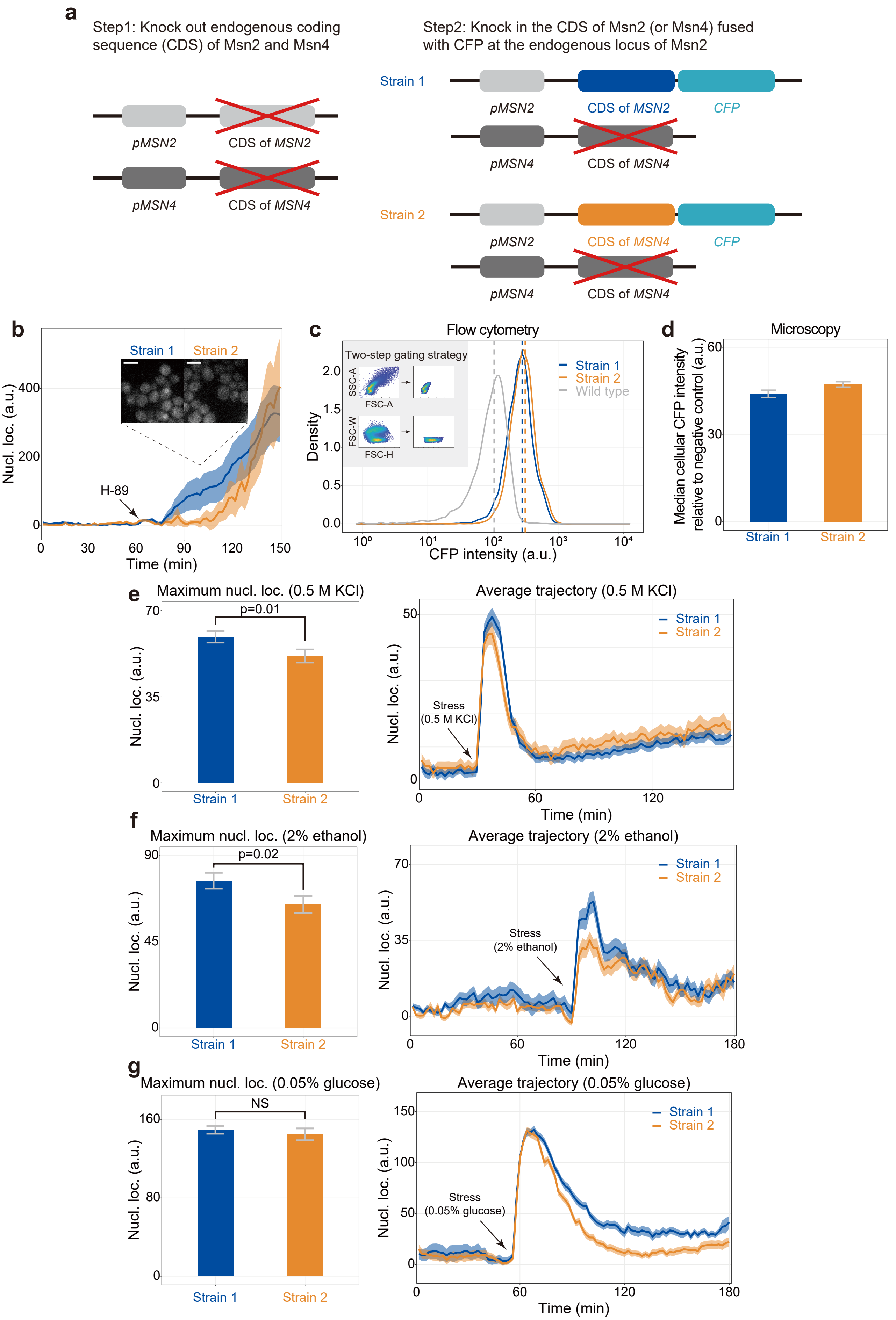

Supplementary Fig. 7: Experimental results suggesting that PKA has a higher affinity for Msn4 than for Msn2. (**a**) Construction of strains for expressing comparable levels of Msn2 versus Msn4. (**b**) Trajectories of nuclear localization responses from the two mutant strains to the sudden addition of PKA inhibitor (300 μM H-89). Cell numbers are 69 and 38 for strain 1 and strain 2, respectively. Example cropped snapshots are shown. Scale bars indicate 5 μm. Shading indicates standard error and centers indicate means. (**c**) CFP intensity distributions (by flow cytometry) of strain 1, strain 2, and the wild type strain (as a control). Cell numbers are 13129, 13145 and 13136 for strain 1, strain 2 and wild type, respectively. Dashed lines indicate means. (**d**) Fluorescence microscopy quantifications of median cellular intensities of Msn2-CFP in strain 1 and Msn4-CFP in strain 2 (after subtracting the background intensity in the wild type strain). Cell numbers are 1142 and 2560 for strain 1 and strain 2, respectively. Error bars indicate standard errors. See also Supplementary Fig. 14g. (**e**) Maximum nuclear localization intensities (see Methods for details) and average trajectory of nuclear localization responses of the two mutant strains to salt stress (0.5 M KCl). One-sided student's t-test was performed. Error bars and shading indicate standard errors and centers indicate means. Cell numbers are 186 and 108 for strain 1 and strain 2, respectively. See also Supplementary Fig. 14h. (**f**) Analogous experiments as in (**e**) for ethanol stress (2% ethanol). One-sided student's t-test was performed. Error bars and shading indicate standard errors and centers indicate means. Cell numbers are 93 and 64. See also Supplementary Fig. 14i. (**g**) Analogous experiments as in (**e**) for glucose limitation stress (0.05% glucose). Note that even though maximum responses were similar between the two strains, Msn2 in strain 1 showed slower nuclear export. Two-sided student's t-test was conducted for maximum nuclear localization. Error bars and shading indicate standard errors and centers indicate means. Cell numbers are 182 and 167 for strain 1 and strain 2, respectively. See also Supplementary Fig. 14j.

Supplementary Fig. 8

a

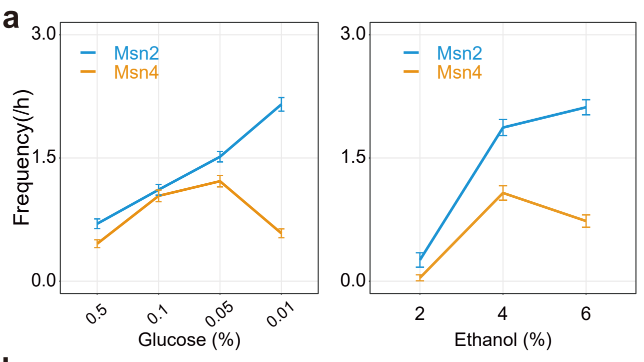

b

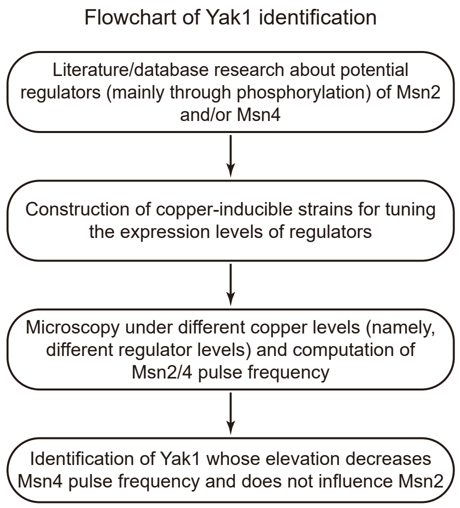

	Reference
CKA2	BioGRID[2]
FPK1	Consensus site, BioGRID
PTK2	BioGRID
RIM15	Lee P, et al., 2013
SNF1	Consensus site, BioGRID
YAK1	Lee P, et al., 2008

c

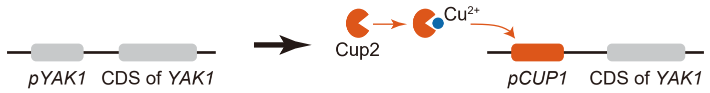

d

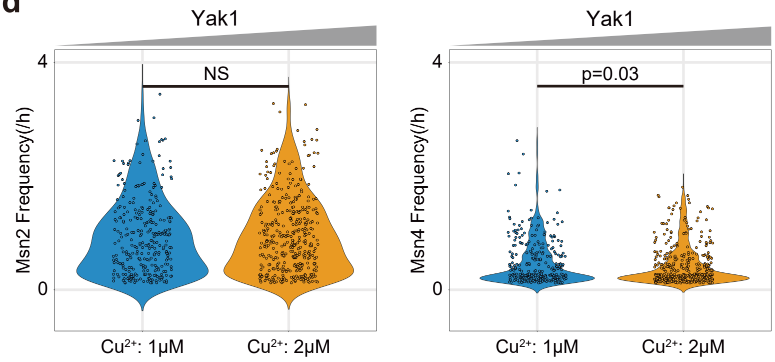

e

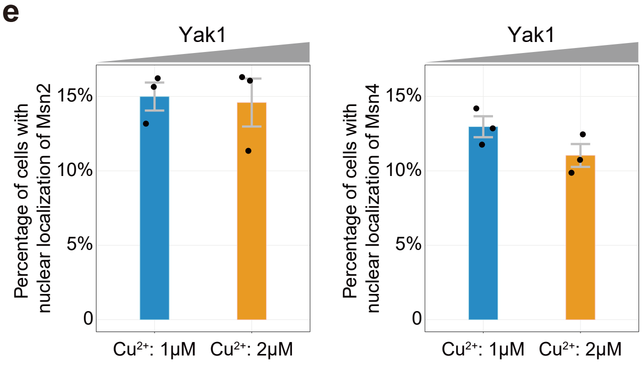

Supplementary Fig. 8: Characterizations of the mechanism underlying temporal redundancy modulation. (**a**) Biphasic dependence of Msn4 pulse frequency on stress level for both glucose limitation stress and ethanol stress. Data was replotted from Supplementary Fig. 1c. Cell numbers are 139, 176, 333, and 282 for glucose limitation stresses, and 54, 159, and 226 for ethanol stresses. Error bars indicate standard errors and centers indicate means. (**b**) Flowchart showing the steps involved in the identification of Yak1 as a candidate for kinase X. **c-e,** Experimental evidence supporting that Yak1 could be the kinase X in our proposed model. (**c**) Endogenous promoter of YAK1 was replaced by copper-inducible *CUP1* promoter for perturbing Yak1 level by adding Cu^{2+} ($CuSO_4$). (**d**) An increase in Yak1 expression level decreases the pulse frequency of Msn4 but not Msn2 under glucose limitation stress (0.05% glucose). Two-sided student's t-test was conducted for Msn2 pulse frequency and one-sided student's t-test was conducted for Msn4 pulse frequency. Cell numbers are 305 and 390 for low and high induction levels, respectively. (**e**) An increase in Yak1 expression level decreases the percentage of cells with nuclear localization of Msn4 but not Msn2 under glucose limitation stress (0.05% glucose). Single time-point snapshots of the mutant strain in (**c**) were used to quantify the nuclear localization of Msn2 and Msn4 under different induction levels. Error bars indicate standard errors of 3 biological repeats and centers indicate means. Cell numbers are 1733 and 1893 for low and high induction levels, respectively.

Supplementary Fig. 9

a

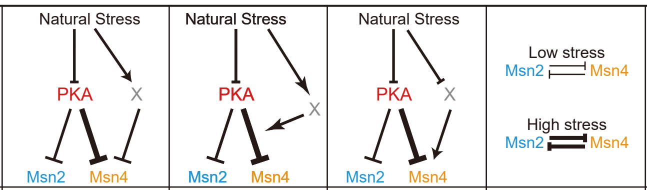

Candidate models					Other models
If compatible with the following evidences: PKA has a higher affinity for Msn4 than for Msn2 (this paper)	√	√	√	Not relevant	
Pulse frequency of Msn4 rather than Msn2 shows biphasic dependence on stress level (this paper)	√	√	√	Not relevant	
Msn4 rather than Msn2 pulses in the absence of PKA[3]	√	×	√	Not relevant	
A kinase inhibiting Msn4 alone is identified (this paper)	√	√	×	Not relevant	
Additional note	(1)		(2)	(3)	(4)

(1) We acknowledge that our model may not be the only model that explains our single-cell data, while it sheds a light into how the divergent temporal dynamics of paralogous TFs could be achieved.
(2) This model is possible if other kinases or phosphatases inducing Msn4 nuclear localization alone are found.
(3) No physical protein-protein interactions between Msn2 and Msn4 are found in the database and since both Msn2 and Msn4 are activated simultaneously and exhibit relatively high levels of nuclear localization under transient responses to stresses, it is also unlikely for Msn2 and Msn4 to negatively affect each other's nuclear localization in an indirect manner.
(4) Other models may also be possible if more evidences are found.

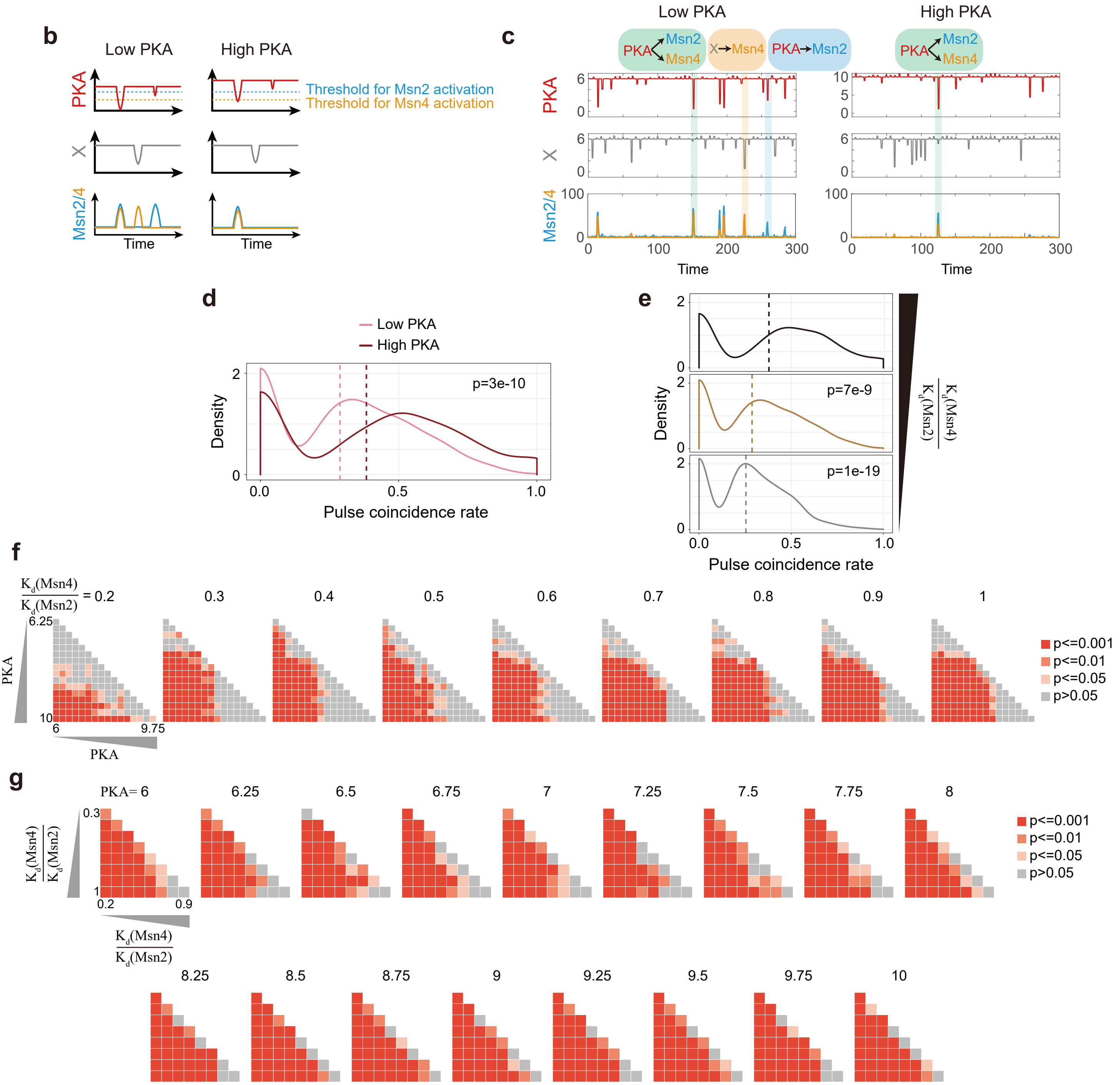

Supplementary Fig. 9: Model predictions for extrinsic and intrinsic controls of temporal relationship between Msn2 and Msn4. (**a**) Discussion about some alternative models. (**b**) Schematics illustrating that pulse coincidence rate increases when elevating PKA. When PKA is elevated, Msn2-only pulse is less likely to occur since the weak down-regulation of PKA can no longer reach the threshold for Msn2 activation. Meanwhile, Msn4-only pulse induced by kinase X is also less likely to occur due to the high activity of PKA and its competition with X to bind with Msn4. (**c**) Example simulated single-cell traces under low PKA (left panel) and high PKA (right panel). Parameters: K_d(Msn4)/K_d(Msn2) = 0.5, PKA is 6 for low PKA and 10 for high PKA. (**d**) Model simulations for the extrinsic control of temporal relationship show that elevating PKA increases pulse coincidence rate and changes its distribution. Cell numbers are 498 and 480 for low (PKA = 7) and high (PKA = 8) PKA. Dashed lines indicate means and the p value of one-sided K-S test is shown. Parameters: K_d(Msn4)/K_d(Msn2) = 0.3. (**e**) Model simulations for the intrinsic control of temporal relationship show that elevating PKA affinity difference (namely, decreasing K_d(Msn4)/K_d(Msn2)) decreases pulse coincidence rate and changes its distribution. Dashed lines indicate means and p values of one-sided K-S test between the bottom two panels and the top panel are shown. Values of K_d(Msn4)/K_d(Msn2) are 0.4, 0.3, and 0.2, and cell numbers are 491, 498, and 500 (top to bottom). Additional parameters: PKA = 7. **f-g,** Elevating PKA (**f**) or decreasing PKA affinity difference (**g**, namely, increasing K_d(Msn4)/K_d(Msn2)) increases pulse coincidence rate and changes its distribution under a wide range of parameter values. For each matrix element, two distributions of pulse coincidence rates were obtained from simulations using parameter values of indicated row or column value (together with the shared parameter indicated on the top of the panel), and one-sided K-S test was performed to obtain the p value. Cell numbers in all conditions are between 271 and 500.

Supplementary Fig. 10

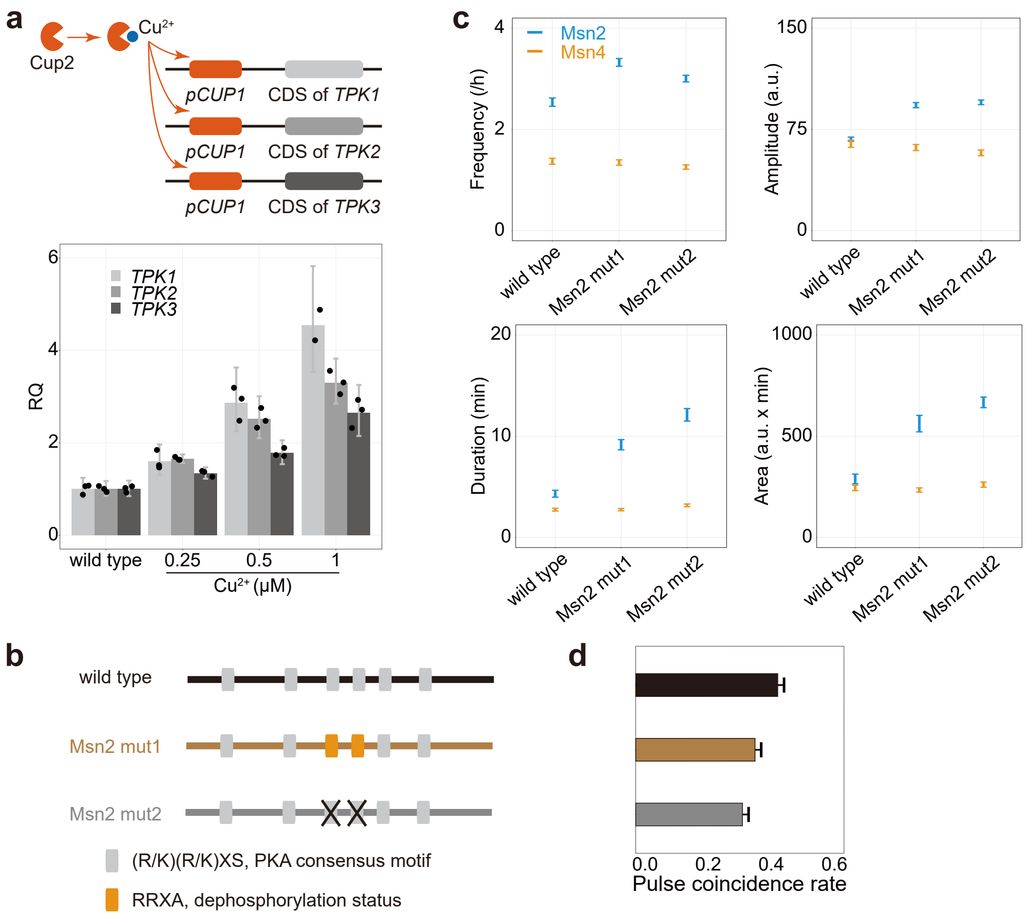

Supplementary Fig. 10: Experimental tests for extrinsic and intrinsic controls of temporal relationship between Msn2 and Msn4. (**a**) Experimental design for testing the extrinsic control of temporal relationship by altering PKA level. Endogenous promoters of *TPK1*, *TPK2* and *TPK3* (three catalytic subunits of PKA) were replaced by copper-inducible *CUP1* promoter and quantitative PCR analysis was performed under different concentrations of Cu^{2+} with wild type serving as the reference. Error bars represent 95% CIs from three technical replicates and centers indicate means. (**b**) Schematic of strains with Msn2 mutations for testing the intrinsic control of temporal relationship. (**c**) Both Msn2 mut1 and Msn2 mut2 increased the frequency, amplitude, duration and area of Msn2 pulse rather than Msn4 pulse under glucose limitation stress (0.01% glucose). Cell numbers are 525, 652, 761, and 752. Error bars indicate standard errors and centers indicate means. (**d**) Both Msn2 mut1 and Msn2 mut2 display decreased pulse coincidence rate between Msn2 and Msn4 relative to wild type under glucose limitation stress (0.01% glucose). Cell numbers are 525, 652, 761, and 752. Error bars indicate 95% CIs and centers indicate means. See also Supplementary Fig. 14k.

Supplementary Fig. 11

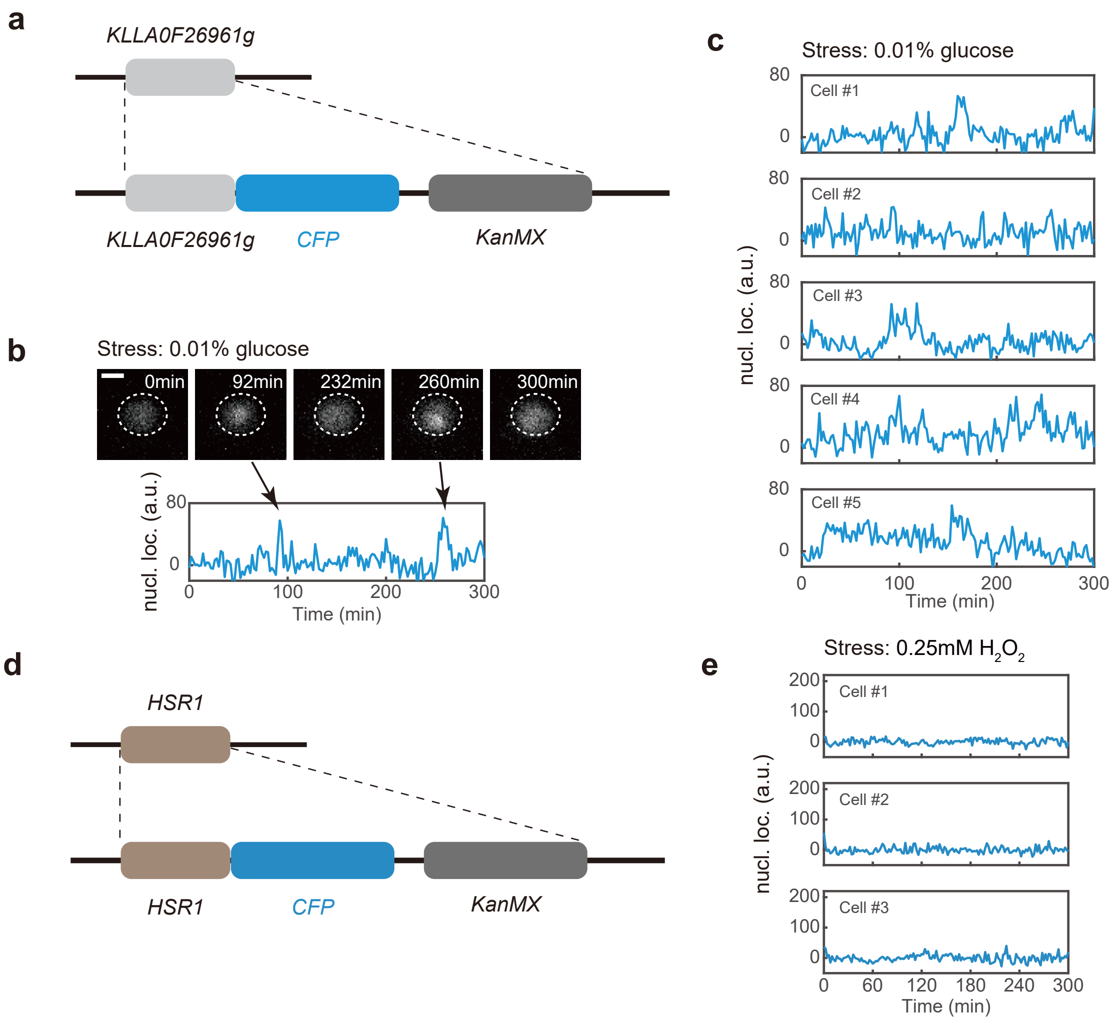

Supplementary Fig. 11: KLLA0F26961g (Msn2/4 ortholog in *K. lactis*) exhibits pulsatile dynamics, while Hsr1 (Msn2/4 ortholog in *S. pombe*) does not. (**a**) Endogenous KLLA0F26961g in *K. lactis* was fused with CFP for imaging its dynamics. (**b**) An example single-cell trace (out of 210 single-cell traces) showing pulsatile dynamics of KLLA0F26961g nuclear localization under glucose limitation stress (0.01% glucose). Filmstrips indicate two pulse events (arrows). White dashed circles indicate cell boundaries and scale bar indicates 2 μm. (**c**) Additional single-cell traces of KLLA0F26961g-CFP nuclear localization showing pulsatile dynamics under glucose limitation (0.01% glucose). See also Supplementary Movie 3. (**d**) Endogenous Hsr1 in *S. pombe* was fused with CFP for imaging its dynamics. (**e**) Hsr1 does not exhibit pulsatile dynamics in *S. pombe*. Three example single-cell traces of Hsr1-CFP nuclear localization are shown under oxidative stress (0.25 mM H_2O_2). See also Supplementary Movie 4.

Supplementary Fig. 12

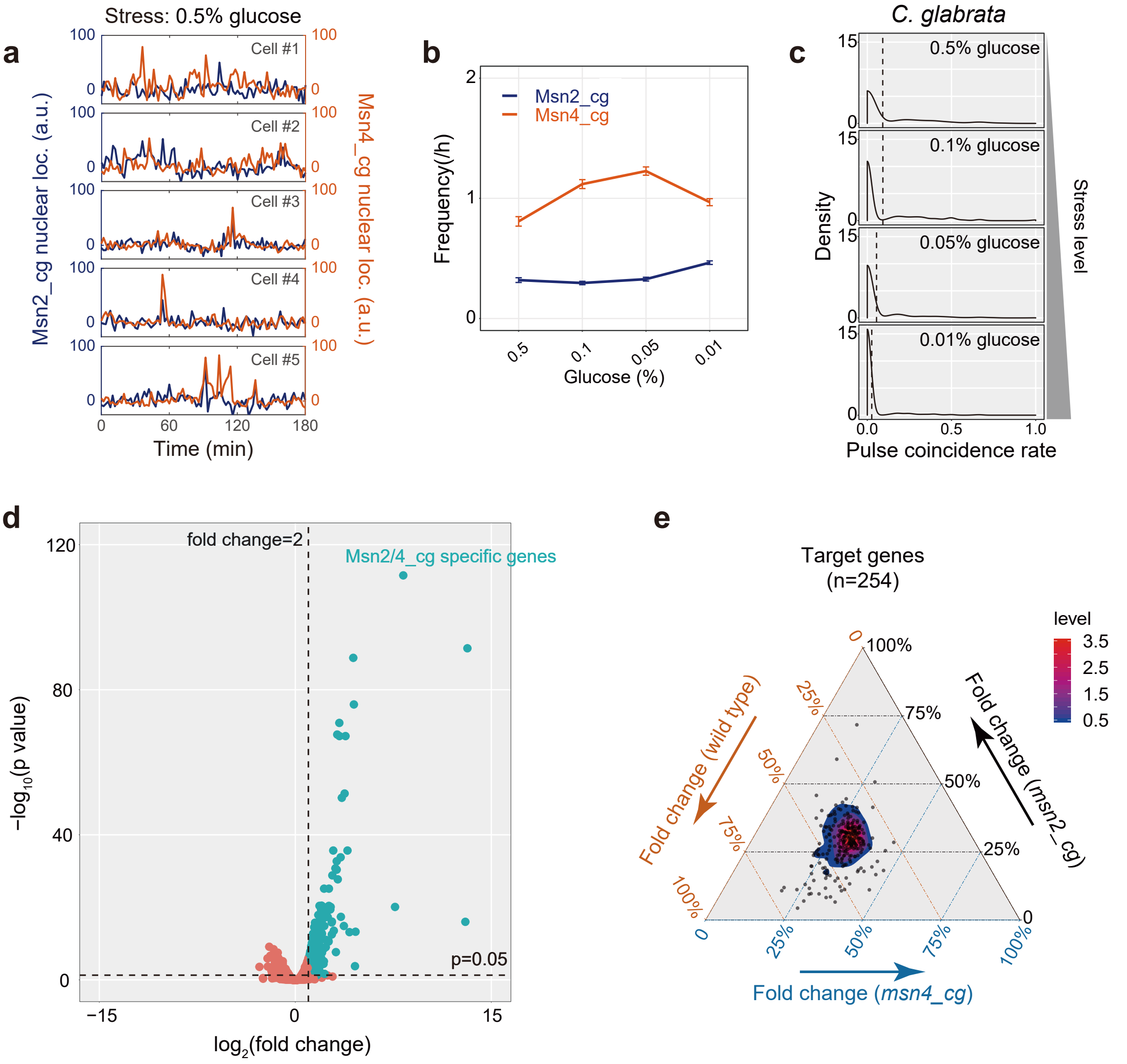

Supplementary Fig. 12: Characterizations of temporal relationship and functional redundancy between Msn2_cg and Msn4_cg in *C. glabrata*. (**a**) Example single-cell traces of Msn2/4_cg nuclear localization dynamics under glucose limitation stress (0.5% glucose) in *C. glabrata*. (**b**) Biphasic dependence of Msn4_cg pulse frequency on the level of glucose limitation stress. Error bars indicate standard errors and centers indicate means (n = 525, 652, 761, and 752 cells, from left to right). (**c**) Distributions of single-cell pulse coincidence rates between Msn2_cg and Msn4_cg under different levels of glucose limitation stress. Dashed lines indicate means. (**d**) Msn2/4_cg target genes (Fig. 5d) were identified by differential gene expression analysis between wild type (*C. glabrata*) and *msn2_cg msn4_cg* under osmotic stress (0.5 M KCl, two biological replicates). 254 genes with fold changes larger than 2 and p values from *DESeq2* software smaller than 0.05 (dashed lines) were identified as Msn2/4_cg specific genes (shown as cyan dots in volcano plot). Note that Msn2_cg and Msn4_cg were also identified but were excluded from the list. (**e**) Results from a biological replicate of Fig. 5d.

Supplementary Fig. 13

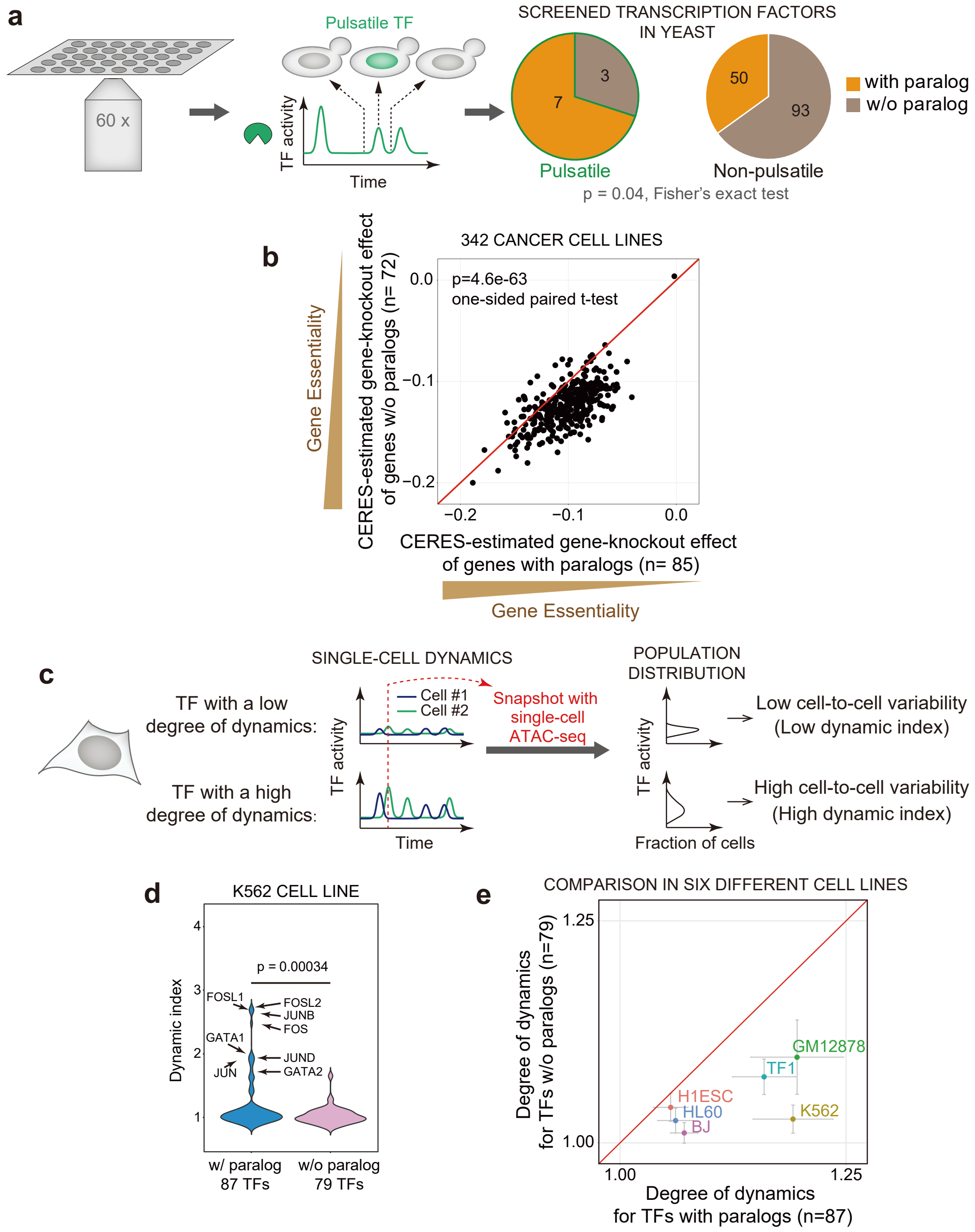

Supplementary Fig. 13: TFs with redundant paralogs are more likely to display temporal activity dynamics than singleton TFs in both yeast and human cells. (**a**) TFs with paralogs are over-represented in pulsatile transcription factors that were identified in a proteome-wide imaging-based screen in yeast[4]. In this assay (left panel), 10 out of 153 yeast TFs were found to be pulsatile (right panel; see middle panel for a schematic of a pulsatile TF). Classification of genes as with or without paralogs was based on Yeast Gene Order Browser[5]. p value of two-sided Fisher's exact test is shown. (**b**) Human TFs with paralogs are less essential than those without paralogs in 342 cancer cell lines, suggesting higher degrees of functional redundancy for paralogous TFs. Data was extracted from CERES-estimated gene-knockout effects in CRISPR–Cas9 essentiality screens[6]. Scores of 0 and −1 correspond to the median effects of nonessential and common core essential genes, respectively. Mean scores of paralogous versus non-paralogous TFs in each cell line were shown as scatter plot. (**c**) Schematic explaining the inference of the degree of TF dynamics (which typically requires time-lapse imaging) based on the cell-to-cell variability of TF activity in the snapshot data of a cell population (e.g., single-cell ATAC-seq data). Note that the ergodicity is assumed. (**d**) Dynamic indexes for paralogous human TFs versus non-paralogous TFs in K562 cell line. Dynamic indexes, or the degrees of dynamics, for TFs were adopted from a previous work[7] that quantified temporal dynamics using single-cell ATAC-seq data of human cell lines. Classification of TFs as either with or without paralogs was based on the literature[8]. p value indicates one-sided student's t-test. (**e**) Dynamic indexes for paralogous human TFs versus non-paralogous TFs in six human cell lines. These results suggest that human TFs with paralogs are more likely to be dynamic than those without paralogs. Error bars indicate standard errors and centers indicate means. n indicates the number of TFs. Note that the set of TFs in (**b**) was a subset of the TFs analyzed in the ATAC-seq data.

Supplementary Fig. 14

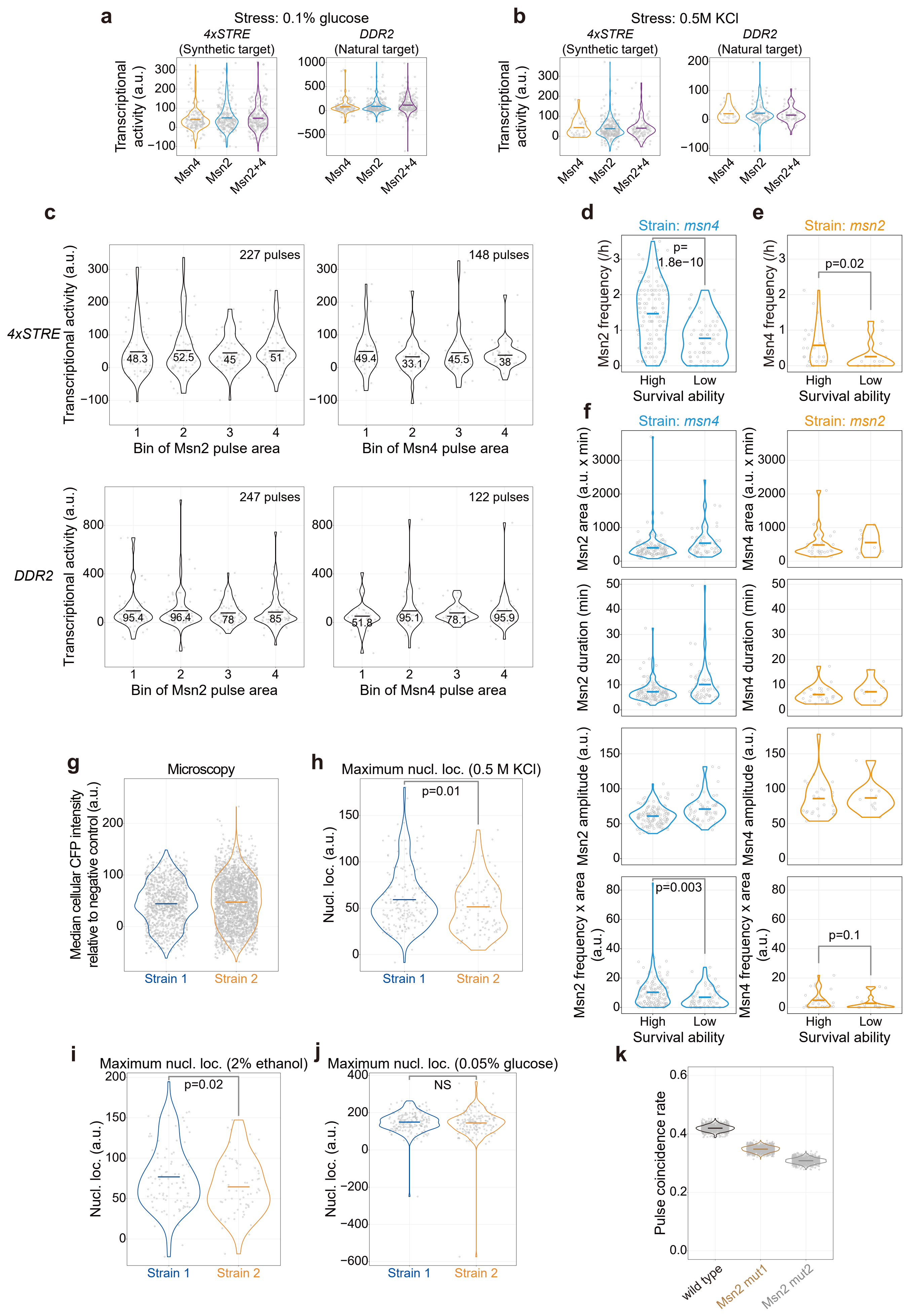

Supplementary Fig. 14: Dot plot presentations of all bar graphs in this study. (**a**) Violin plot presentation of Fig. 2b. Short horizontal lines indicate means. (**b**) Violin plot presentation of Supplementary Fig. 4a. Short horizontal lines indicate means. (**c**) Violin plot presentation of Supplementary Fig. 4d. Short horizontal lines and numbers indicate means. **d-e,** Violin plot presentations of Supplementary Fig. 6a-b. Short horizontal lines indicate means and p values indicate one-sided student's t-test. (**f**) Violin plot presentation of Supplementary Fig. 6c. Short horizontal lines indicate means and p values indicate one-sided student's t-test. (**g**) Violin plot presentation of Supplementary Fig. 7d. Short horizontal lines indicate means. (**h**) Violin plot presentation of Supplementary Fig. 7e. Short horizontal lines indicate means and p value indicates one-sided student's t-test. (**i**) Violin plot presentation of Supplementary Fig. 7f. Short horizontal lines indicate means and p value indicates one-sided student's t-test. (**j**) Violin plot presentation of Supplementary Fig. 7g. Short horizontal lines indicate means and two-sided student's t-test was conducted. (**k**) Violin plot presentation of Supplementary Fig. 10d. Short horizontal lines indicate means. Dot numbers of all figures are indicated in the legends of original figures.

Supplementary Table 1. List of strains constructed and used in this study

ID	Strain designation	Strain genotype	Species	Used in figure
7	*MSN2-CFP MSN4-YFP*	*BY4741 MSN2-CFP::spHIS5 MSN4-YFP::CaURA3*	*Saccharomyces cerevisiae*	3c-e; S5a-c, S6d-e;
25	*MSN2-CFP MSN4-YFP PCP-iRFP 4xSTRE-24xPP7*	*BY4741 MSN2-CFP::spHIS5 MSN4-YFP::CaURA3 PCP-iRFP::LEU2 4xSTRE-24xPP7::KANMX*	*Saccharomyces cerevisiae*	1b-d; 2a-c; 3b; 4d; S1c; S2-4; S8a; S10; S14a-c, k;
48	*msn2 MSN4-YFP*	*BY4741 msn2::MET17 MSN4-YFP::CaURA3*	*Saccharomyces cerevisiae*	S6b-c; S14e-f;
49	*msn4 MSN2-CFP*	*BY4741 MSN2-CFP::spHIS5 msn4::MET17*	*Saccharomyces cerevisiae*	S6a, c; S14d, f;
55	*MSN2-CFP MSN4-YFP PCP-iRFP DDR2-24xPP7*	*BY4741 MSN2-CFP::spHIS5 MSN4-YFP::CaURA3 PCP-iRFP::LEU2 DDR2-24xPP7::KANMX*	*Saccharomyces cerevisiae*	2b; S4a, d; S14a-c;
63	*msn2 MSN4-YFP PCP-iRFP 4xSTRE-24xPP7*	*BY4741 msn2::MET17 MSN4-YFP::CaURA3 PCP-iRFP::LEU2 4xSTRE-24xPP7::KANMX*	*Saccharomyces cerevisiae*	2c; S4c;
66	*MSN2-CFP msn4 PCP-iRFP 4xSTRE-24xPP7*	*BY4741 MSN2-CFP::spHIS5 msn4::MET17 PCP-iRFP::LEU2 4xSTRE-24xPP7::KANMX*	*Saccharomyces cerevisiae*	2c; S4c;
126	*MSN2-CFP msn4 4xSTRE-YFP*	*BY4741 MSN2-CFP::spHIS5 msn4::MET17 4xSTRE-YFP(yEVenus)::KANMX*	*Saccharomyces cerevisiae*	S7; S14g-j;
128	*MSN2p-MSN4-CFP msn4 4xSTRE-YFP*	*BY4741 MSN2::MSN4-CFP::spHIS5 msn4::MET17 4xSTRE-YFP(yEVenus)::KANMX*	*Saccharomyces cerevisiae*	S7; S14g-j;
175	*MSN2-CFP MSN4-YFP CUP1p-TPK1 CUP1p-TPK2 CUP1p-TPK3*	*BY4741 MSN2-CFP::spHIS5 MSN4-YFP::CaURA3 TPK1p::LEU2-CUP1p-TPK1 TPK2p::KANMX-CUP1p-TPK2 TPK3p::MET17-CUP1p-TPK3*	*Saccharomyces cerevisiae*	4c; S10a;
183	*HSR1-CFP*	*972h- HSR1-CFP::KANMX*	*Schizosaccharomyces pombe*	S11e;

184	*KLLA0F26961g-CFP*	*CBS141 KLLA0F26961g-CFP::KANMX*	*Kluyveromyces lactis*	S11b-c;
189	*BG2*	*BG2(wild type)*	*Candida glabrata*	5d; S12d-e;
193	*MSN2_cg-CFP MSN4_cg-YFP*	*BG2 CAGL0F05995g-CFP::KANMX CAGL0M13189g-YFP::NATMX6*	*Candida glabrata*	5b-c; S12a-c;
195	*msn2_cg*	*BG2 cagl0f05995g::KANMX*	*Candida glabrata*	5d; S12e;
196	*msn4_cg*	*BG2 cagl0m13189g::NATMX6*	*Candida glabrata*	5d; S12e;
197	*msn2_cg msn4_cg*	*BG2 cagl0f05995g::KANMX cagl0m13189g::NATMX6*	*Candida glabrata*	S12d;
198	*MSN2 mut2*	*BY4741 MSN2(Δaa619-629::PVQPRK)-CFP::spHIS5 MSN4-YFP::CaURA3 PCP-iRFP::LEU2 4xSTRE-24xPP7::KANMX*	*Saccharomyces cerevisiae*	4d; S10c-d; S14k;
202	*MSN2 mut1*	*BY4741 MSN2(Δaa617-620::RRPA, Δaa622-625::RRKA)-CFP::spHIS5 MSN4-YFP::CaURA3 PCP-iRFP::LEU2 4xSTRE-24xPP7::KANMX*	*Saccharomyces cerevisiae*	4d; S10c-d; S14k;
208	*MSN2(ΔDBD)-CFP MSN4(ΔDBD)-YFP*	*BY4741 MSN2(Δaa642-704)-CFP::spHIS5 MSN4(Δaa568-630)-YFP::LEU2*	*Saccharomyces cerevisiae*	S5d-e;
211	*MSN2-CFP MSN4-YFP CUP1p-YAK1*	*BY4741 MSN2-CFP::spHIS5 MSN4-YFP::CaURA3 YAK1p::LEU2-CUP1p-YAK1*	*Saccharomyces cerevisiae*	S8d-e;

Supplementary Table 2. List of primers used in this study

Name	Sequence (5’ to 3’)
MSN2-fusion-upstream-homology-arm-F	gtacggtgccgcttacaaat
MSN2-fusion-upstream-homology-arm-R	aatgtctccatgttttttatgagtct
MSN2-fusion-downstream-homology-arm-F	tagaccccattttttaattcg
MSN2-fusion-downstream-homology-arm-R	ataatgcgcacggaattcat
MSN4-fusion-upstream-homology-arm-F	gctaaattggcgacttctgg
MSN4-fusion-upstream-homology-arm-R	aaaatcaccgtgctttttgtg
MSN4-fusion-downstream-homology-arm-F	aaaaaataaggtcaaaagcaaataaaagc
MSN4-fusion-downstream-homology-arm-R	agcaaacgtcgtaccaatcc
MSN2-deletion-upstream-homology-arm-F	ttccagcgaaagagacaggt
MSN2-deletion-upstream-homology-arm-R	tcatggtcgaccgtcatt
MSN2-deletion-downstream-homology-arm-F	tagaccccattttttaattcgatagatctt
MSN2-deletion-downstream-homology-arm-R	ataatgcgcacggaattcat
MSN4-deletion-upstream-homology-arm-F	ctttctcccacgaggtttca
MSN4-deletion-upstream-homology-arm-R	tgcgtgacgaacgaaactactattag
MSN4-deletion-downstream-homology-arm-F	aaaaaataaggtcaaaagcaaataaaagcaaga
MSN4-deletion-downstream-homology-arm-R	agcaaacgtcgtaccaatcc
DDR2-fusion-upstream-homology-arm-F	caaggtgtcaaacatcaataaa
DDR2-fusion-upstream-homology-arm-R	ctaaatcaaaaaggccaaagca
DDR2-fusion-downstream-homology-arm-F	gaaaaacgccgcttactgccac
DDR2-fusion-downstream-homology-arm-R	ctcacatgcattacacctttggtttc
MSN4-CDS-F	atgctagtcttcggacctaatag
MSN4-CDS-R	aaaatcaccgtgctttttgtgag
CUP1-promoter-F	ctagttagaaaaagacatttttgctgtcagtcac
CUP1-promoter-R	tttatgtgatgattgattgattgattgtacagttt
TPK1-promoter-replacement-upstream-homology-arm-F	gatagacaagatagacatcttttgcatacagtttt
TPK1-promoter-replacement-upstream-homology-arm-R	atgtttttaaggcccgtccagcg
TPK1-promoter-replacement-downstream-homology-arm-F	atgtcgactgaagaacaaaatggag
TPK1-promoter-replacement-downstream-homology-arm-R	atataatccataatcatgaaaatttgctgagcat
TPK2-promoter-replacement-upstream-homology-arm-F	ccagaagagatcaaacaacaaactatcttgaa

TPK2-promoter-replacement-upstream-homology-arm-R	atatacatatgtttatttttgtgcgttttgagcaat
TPK2-promoter-replacement-downstream-homology-arm-F	atggaattcgttgcagaaagggc
TPK2-promoter-replacement-downstream-homology-arm-R	ttaggaaatctttgtgactttctcagtaacg
TPK3-promoter-replacement-upstream-homology-arm-F	atgtcccaactatttcaaaagttgtacatg
TPK3-promoter-replacement-upstream-homology-arm-R	agagtatctgaaacgttgacgcaatt
TPK3-promoter-replacement-downstream-homology-arm-F	atgtatgttgatccgatgaacaacaatgaaa
TPK3-promoter-replacement-downstream-homology-arm-R	tagtccattaccatgaaaacttgctgagaat
HSR1-fusion-upstream-homology-arm-F	aaagccatttgtttgtatatgtggaaaacg
HSR1-fusion-upstream-homology-arm-R	agaactaggataaggttgatattgatgcatcg
HSR1-fusion-downstream-homology-arm-F	gcatttttataaattaactgcctaaattcgggtttttt
HSR1-fusion-downstream-homology-arm-R	ttcactgtacattagtttattggatttgtaaaaccgt
KLLA0F26961g-fusion-upstream-homology-arm-F	aggctcagaagaaaatctgcattgaatagg
KLLA0F26961g-fusion-upstream-homology-arm-R	gtgcttgtttgaagaattcgtgactct
KLLA0F26961g-fusion-downstream-homology-arm-F	aataatctacgatatttcagatgtgacattggaaatgag
KLLA0F26961g-fusion-downstream-homology-arm-R	aagccgaaacgtacctttctattatctttgt
MSN2_cg-fusion-upstream-homology-arm-F	gcaaaccaaaacttctacaaccctgaaa
MSN2_cg-fusion-upstream-homology-arm-R	ttctttagttgtggaagtacttcccgact
MSN2_cg-fusion-downstream-homology-arm-F	tgacagtgttccttattttatctagttggcg
MSN2_cg-fusion-downstream-homology-arm-R	tagcaatgaaaagacaaattctcgttaccgc
MSN4_cg-fusion-upstream-homology-arm-F	gaagatgctgtcctgtccgatgatga
MSN4_cg-fusion-upstream-homology-arm-R	aaagtctccgtgtcttttatgtgttttcaaat
MSN4_cg-fusion-downstream-homology-arm-F	ttaaagtctcgagccattctttattttattttctgct
MSN4_cg-fusion-downstream-homology-arm-R	tcgggggcatctaataattttccgtttca
MSN2_cg-deletion-upstream-homology-arm-F	ccgtttactcaacaatgagacgcaagtcc
MSN2_cg-deletion-upstream-homology-arm-R	ctgttcttgttgatctgtgtttggtatggcc
MSN2_cg-deletion-downstream-homology-arm-F	tgacagtgttccttattttatctagttggcg
MSN2_cg-deletion-downstream-homology-arm-R	tagcaatgaaaagacaaattctcgttaccgc
MSN4_cg-deletion-upstream-homology-arm-F	cccaaatgcagagaacatgttccatgcc

MSN4_cg-deletion-upstream-homology-arm-R	attggcaagtcttgtttattttttactttcaacttttattgtgtatgttt
MSN4_cg-deletion-downstream-homology-arm-F	ttaaagtctcgagccattctttattttattttctgct
MSN4_cg-deletion-downstream-homology-arm-R	tcgggggcatctaataattttccgtttca
MSN2_mut2-part1-F	ttccagcgaaagagacaggt
MSN2_mut2-part1-R	cttttccgaggttggacaggccttctttccttcgtaaccccagcac
MSN2_mut2-part2-F	cctgtccaacctcggaaaagaagatcaagtgtcgtaatagaatcaacaaaggaact
MSN2_mut2-part2-R	aatgtctccatgttttttatgagtct
MSN2_mut1-part1-F	ttccagcgaaagagacaggtaataaagacaaatagcag
MSN2_mut1-part1-R	attacgacacttgatcttctggacggtgtcatagcttttctcctgtaagctggccttctttccttcgtaaccccagcac
MSN2_mut1-part2-F	agaagatcaagtgtcgtaatagaatcaacaaaggaactcg
MSN2_mut1-part2-R	aatgtctccatgttttttatgagtct
MSN2DBD-deletion-upstream-homology-arm-F	gatctttattcaagaaggcaaagagcttctttaccc
MSN2DBD-deletion-upstream-homology-arm-R	ttcctttgttgattctattacgacacttgatcttctgg
MSN2DBD-deletion-downstream-homology-arm-F	tagaccccattttttaattcgatagatctttcttcataagataattctg
MSN2DBD-deletion-downstream-homology-arm-R	ataatgcgcacggaattcattaaactgtacggatat
MSN4DBD-deletion-upstream-homology-arm-F	aacaaattatccctgaaggtactgccactac
MSN4DBD-deletion-upstream-homology-arm-R	gtagttgttggggtcaatggtagtaatagatttcctt
MSN4DBD-deletion-downstream-homology-arm-F	aaaaaataaggtcaaaagcaaataaaagcaagacaagct
MSN4DBD-deletion-downstream-homology-arm-R	agcaaacgtcgtaccaatccttgaatgc
YAK1-promoter-replacement-upstream-homology-arm-F	atcatcagcgttgacgtcatcttcatcc
YAK1-promoter-replacement-upstream-homology-arm-R	gttgagtttgagggcgaaagtacacaaa
YAK1-promoter-replacement-downstream-homology-arm-F	atgaactcatccaataataacgactcgtccag
YAK1-promoter-replacement-downstream-homology-arm-R	gaaggatactggccactgctatttcctg
TPK1-qPCR-F	taaggacactgggtacgggt
TPK1-qPCR-R	ggcgtgtatcctgctagcat
TPK2-qPCR-F	tagggttcatttggtgcgct

TPK2-qPCR-R	gaccaccaatccaccgactt
TPK3-qPCR-F	gggaactggctcatttggga
TPK3-qPCR-R	agcacaccaaaactccacca
ACT1-qPCR-F	tcccaggtattgccgaaagaatgc
ACT1-qPCR-R	gccaagatagaaccaccaatccaga

Supplementary Table 3. Details of reagents used in this study

Reagent	Company	Catalog number
Yeast extract	OXOID	LP0021
Bacto peptone	BD	211677
Glucose	SIGMA	V900392-500G
Yeast nitrogen base w/o amino acids	BD	291940
Amino acid drop-out mix	Clontech/MP	630423/114550412-CF
EMM2 media	ELITE-MEDIA	M886-01
DRAQ7 dye	Abcam	ab109202
Concanavalin A	SIGMA	C2010-250MG
Potassium chloride	SIGMA	P9541-500G
Hydrogen peroxide	SIGMA	88597-100ML-F
H-89	MCE	HY-15979A
$CuSO_4$	SIGMA	C1297-100G
L-Leucine	SIGMA	61819-25G
L-Histidine	SIGMA	53319-25G
Uracil	SIGMA	U0750-5G
L-Methionine	SIGMA	M9625-5G
L-Tryptophan	SIGMA	T0254-1G
Nourseothricin Sulfate	HARVEYBIO	N21030-100MG
Geneticin	Gibco	10131-035
Frozen-EZ Yeast Transformation II Kit	Zymo Research	T2001
HiPure HP Plant RNA Mini Kit	Magen	R4165-01
iScript	Bio-Rad	1708890
FastKing RT Kit	Tiangen	KR116-01
GoTaq qPCR Master Mix	Promega	A6001

References

1. Zhang, R. *et al.* High-throughput single-cell analysis for the proteomic dynamics study of the yeast osmotic stress response. *Sci Rep* **7**, 42200 (2017).
2. Oughtred, R. *et al.* The BioGRID database: A comprehensive biomedical resource of curated protein, genetic, and chemical interactions. *Protein Sci* **30**, 187-200 (2021).
3. Jacquet, M., Renault, G., Lallet, S., De Mey, J. & Goldbeter, A. Oscillatory nucleocytoplasmic shuttling of the general stress response transcriptional activators Msn2 and Msn4 in Saccharomyces cerevisiae. *J Cell Biol* **161**, 497-505 (2003).
4. Dalal, C.K., Cai, L., Lin, Y.H., Rahbar, K. & Elowitz, M.B. Pulsatile Dynamics in the Yeast Proteome. *Curr Biol* **24**, 2189-2194 (2014).
5. Byrne, K.P. & Wolfe, K.H. The Yeast Gene Order Browser: combining curated homology and syntenic context reveals gene fate in polyploid species. *Genome Res* **15**, 1456-1461 (2005).
6. Meyers, R.M. *et al.* Computational correction of copy number effect improves specificity of CRISPR-Cas9 essentiality screens in cancer cells. *Nature Genetics* **49**, 1779-+ (2017).
7. Buenrostro, J.D. *et al.* Single-cell chromatin accessibility reveals principles of regulatory variation. *Nature* **523**, 486-490 (2015).
8. Dandage, R. & Landry, C.R. Paralog dependency indirectly affects the robustness of human cells. *Molecular Systems Biology* **15** (2019).
