# Supplementary material for: Yeast cell fate control by temporal redundancy modulation of transcription factor paralogs
Source: Nat Commun. 2021 May 25;12:3145. doi: 10.1038/s41467-021-23425-0 (PMC8149833; doi:10.1038/s41467-021-23425-0)
Supplement: Supplementary file 7 — Description of additional supplementary files [file 41467_2021_23425_MOESM7_ESM.docx]

Description of additional supplementary information

Title: Supplementary Movie 1.

Description: Single-cell movie of Msn2-CFP and Msn4-YFP dynamics in response to glucose limitation stress. The movie and the corresponding nuclear localization traces of the cell in Fig. 1b are shown. The switch in glucose (from 2% to 0.1%) is indicated by the arrow.

Title: Supplementary Movie 2.

Description: Single-cell movie of Msn2/4 nuclear localization dynamics and DRAQ7 signal under glucose limitation stress. The movie and the corresponding traces of Msn2/4 nuclear localization and DRAQ7 signal of the cell in Fig. 3c are shown.

Title: Supplementary Movie 3.

Description: KLLA0F26961g (ortholog of Msn2 and Msn4 in K. lactis) shows pulsatile dynamics. The movie shows the time-lapse images of CFP-fused KLLA0F26961g under glucose limitation stress (0.01% glucose). Duration of the movie is 300 minutes and scale bar indicates 2 μm.

Title: Supplementary Movie 4.

Description: Hsr1 (ortholog of Msn2/4 in S. pombe) shows no dynamics. The movie shows the time-lapse images of CFP-fused Hsr1 under hydrogen peroxide (0.25 mM) stress. Duration of the movie is 300 minutes and scale bar indicates 5 μm
