# Supplementary material for: Yeast cell fate control by temporal redundancy modulation of transcription factor paralogs
Source: Nat Commun. 2021 May 25;12:3145. doi: 10.1038/s41467-021-23425-0 (PMC8149833; doi:10.1038/s41467-021-23425-0)
Supplement: Supplementary file 8 — Reporting Summary [file 41467_2021_23425_MOESM8_ESM.pdf]

nature research

Corresponding author(s): Yihan Lin

Last updated by author(s): Mar 13, 2021

Reporting Summary

Nature Research wishes to improve the reproducibility of the work that we publish. This form provides structure for consistency and transparency in reporting. For further information on Nature Research policies, see our Editorial Policies and the Editorial Policy Checklist.

Statistics

For all statistical analyses, confirm that the following items are present in the figure legend, table legend, main text, or Methods section.

n/a	Confirmed	
☐	☒	The exact sample size (*n*) for each experimental group/condition, given as a discrete number and unit of measurement
☐	☒	A statement on whether measurements were taken from distinct samples or whether the same sample was measured repeatedly
☐	☒	The statistical test(s) used AND whether they are one- or two-sided<br>*Only common tests should be described solely by name; describe more complex techniques in the Methods section.*
☒	☐	A description of all covariates tested
☒	☐	A description of any assumptions or corrections, such as tests of normality and adjustment for multiple comparisons
☐	☒	A full description of the statistical parameters including central tendency (e.g. means) or other basic estimates (e.g. regression coefficient) AND variation (e.g. standard deviation) or associated estimates of uncertainty (e.g. confidence intervals)
☐	☒	For null hypothesis testing, the test statistic (e.g. *F*, *t*, *r*) with confidence intervals, effect sizes, degrees of freedom and *P* value noted<br>*Give P values as exact values whenever suitable.*
☒	☐	For Bayesian analysis, information on the choice of priors and Markov chain Monte Carlo settings
☒	☐	For hierarchical and complex designs, identification of the appropriate level for tests and full reporting of outcomes
☒	☐	Estimates of effect sizes (e.g. Cohen's *d*, Pearson's *r*), indicating how they were calculated

Our web collection on statistics for biologists contains articles on many of the points above.

Software and code

Policy information about availability of computer code

Data collection	Micro-Manager v1.4; Custom scripts are available at https://github.com/wyzfcb/code_of_yeast_cell_fate_control_paper.
Data analysis	CircularHough_GrdEx function from Mathworks File Exchange; 7500 software v2.3 (Applied Biosystems); Trimmomatic v0.38; Tophat v2.1.1; Cufflinks v2.2.1; featureCounts v1.6.3; R package DESeq2 v1.28.1; R package clusterProfiler v3.16.1; EasyFlow (https://antebilab.github.io/easyflow); Custom scripts are available at https://github.com/wyzfcb/code_of_yeast_cell_fate_control_paper.

For manuscripts utilizing custom algorithms or software that are central to the research but not yet described in published literature, software must be made available to editors and reviewers. We strongly encourage code deposition in a community repository (e.g. GitHub). See the Nature Research guidelines for submitting code & software for further information.

Data

Policy information about availability of data

All manuscripts must include a data availability statement. This statement should provide the following information, where applicable:

- Accession codes, unique identifiers, or web links for publicly available datasets
- A list of figures that have associated raw data
- A description of any restrictions on data availability

All sequencing data generated in this study have been deposited in publicly accessible databases. The processed and raw RNA-seq data are available in the NCBI Gene Expression Omnibus (GEO) database under the accession code GSE161373 [https://www.ncbi.nlm.nih.gov/geo/query/acc.cgi?acc=GSE161373]. Source data are provided with this paper. The data that support the findings of this study are available from the corresponding author upon reasonable request.

Field-specific reporting

Please select the one below that is the best fit for your research. If you are not sure, read the appropriate sections before making your selection.

☒ Life sciences ☐ Behavioural & social sciences ☐ Ecological, evolutionary & environmental sciences

For a reference copy of the document with all sections, see nature.com/documents/nr-reporting-summary-flat.pdf

Life sciences study design

All studies must disclose on these points even when the disclosure is negative.

Sample size	No sample size calculation was performed. Sample size is the number of single cells/pulses after microscopy and in the field of single-cell study, sample size larger than 50 is typically sufficient.
Data exclusions	Only cells with tracking errors or at the corners of the imaging field during time-lapse microscopy were discarded due to low quality.
Replication	We conducted >=2 biologically independent experiments to obtain more than ~50 single cells. All attempts at replication were successful.
Randomization	This is not relevant to our study since our samples are single cells from a single colony.
Blinding	Blinding is not relevant to our study since our samples are single cells from a single colony.

Reporting for specific materials, systems and methods

We require information from authors about some types of materials, experimental systems and methods used in many studies. Here, indicate whether each material, system or method listed is relevant to your study. If you are not sure if a list item applies to your research, read the appropriate section before selecting a response.

Materials & experimental systems

n/a	Involved in the study	
☒	☐	Antibodies
☒	☐	Eukaryotic cell lines
☒	☐	Palaeontology and archaeology
☒	☐	Animals and other organisms
☒	☐	Human research participants
☒	☐	Clinical data
☒	☐	Dual use research of concern

Methods

n/a	Involved in the study	
☒	☐	ChIP-seq
☐	☒	Flow cytometry
☒	☐	MRI-based neuroimaging

Flow Cytometry

Plots

Confirm that:

☒ The axis labels state the marker and fluorochrome used (e.g. CD4-FITC).

☒ The axis scales are clearly visible. Include numbers along axes only for bottom left plot of group (a 'group' is an analysis of identical markers).

☒ All plots are contour plots with outliers or pseudocolor plots.

☒ A numerical value for number of cells or percentage (with statistics) is provided.

Methodology

Sample preparation	For flow cytometry experiments, overnight single-colony culture was diluted to OD600 = 0.005. After 4-hour culture with normal media, fluorescent intensity of CFP was measured.
Instrument	BD LSRFortessa flow cytometer
Software	EasyFlow (https://antebilab.github.io/easyflow)
Cell population abundance	This is not relevant to our study since our samples are single yeast cells from the same single colony, which indicates that they are genetically identical and pure. We just gated for singlets and the abundance is indicated in the legend.

Gating strategy

We only gated for singlets and the gating strategy is provided as an insert in the related figure.

☒ Tick this box to confirm that a figure exemplifying the gating strategy is provided in the Supplementary Information.
